# Validation of a direct-to-PCR COVID-19 detection protocol utilizing mechanical homogenization: A model for reducing resources needed for accurate testing

**DOI:** 10.1371/journal.pone.0256316

**Published:** 2021-08-18

**Authors:** Zachary P. Morehouse, Lyson Samikwa, Caleb M. Proctor, Harry Meleke, Mercy Kamdolozi, Gabriella L. Ryan, David Chaima, Antonia Ho, Rodney J. Nash, Tonney S. Nyirenda

**Affiliations:** 1 Michigan State University College of Osteopathic Medicine, East Lansing, Michigan, United States of America; 2 Omni International Inc, A PerkinElmer Company, Kennesaw, Georgia, United States of America; 3 Jeevan BioSciences, Tucker, Georgia, United States of America; 4 Department of Pathology, College of Medicine, University of Malawi, Blantyre, Malawi; 5 MRC-University of Glasgow Centre for Virus Research, Glasgow, United Kingdom; 6 Department of Biology, Georgia State University, Atlanta, Georgia, United States of America; Waseda University: Waseda Daigaku, JAPAN

## Abstract

Efficient and effective viral detection methodologies are a critical piece in the global response to COVID-19, with PCR-based nasopharyngeal and oropharyngeal swab testing serving as the current gold standard. With over 100 million confirmed cases globally, the supply chains supporting these PCR testing efforts are under a tremendous amount of stress, driving the need for innovative and accurate diagnostic solutions. Herein, the utility of a direct-to-PCR method of SARS-CoV-2 detection grounded in mechanical homogenization is examined for reducing resources needed for testing while maintaining a comparable sensitivity to the current gold standard workflow of nasopharyngeal and oropharyngeal swab testing. In a head-to-head comparison of 30 patient samples, this initial clinical validation study of the proposed homogenization-based workflow demonstrated significant agreeability with the current extraction-based method utilized while cutting the total resources needed in half.

## Introduction

The COVID-19 global pandemic caused by SARS-CoV-2 has dominated the clinical and research landscapes since its designation by the World Health Organization in March of 2020 [1]. Stretching to all corners of the globe, SARS-CoV-2 has been detected in most communities causing activation of public health efforts geared towards disease surveillance and prevention practices [2–4]. Efficient and effective SARS-CoV-2 detection methods are the cornerstone of these public health efforts [2–4]. Following the common detection method of many other positive-sense RNA respiratory viruses, reverse transcriptase polymerase chain reaction (RT-PCR) testing quickly emerged as the global standard for accurate SARS-CoV-2 detection from patient samples [3, 4]. Due to their high sensitivity for viral detection, nasopharyngeal and oropharyngeal swabs are widely used sample types obtained from patients for the purpose of RT-PCR based COVID-19 testing [3].

In the traditional model of RT-PCR viral testing, swab samples are collected from the patient, stored in viral transport media at the collection site, and sent to the laboratory for processing via molecular techniques including viral RNA extraction and purification utilizing a series of chemical reagents followed by RT-PCR for viral detection [3, 5, 6]. While proven effective, this traditional model requires a significant quantity of consumable plastics and chemical reagents to complete, particularly in the extraction and purification steps that include multiple rounds of digestions and different buffer washes [7]. As the COVID-19 pandemic spread, the international supply of the common reagents and plastics used in viral genetic extractions and RT-PCR testing became increasingly stressed reducing access to adequate testing and driving up prices to health systems and patients [7].

With the demand for testing increasing secondary to viral spread throughout communities, the need for cost effective and accurate diagnostic capabilities drove the innovation of novel viral detection methodologies [3, 4, 6, 7]. One way to reduce cost and increase access to SARS-CoV-2 testing is to reduce the resources needed for detection, without reducing the sensitivity of the testing assays. Technologies such as isothermal PCR reactions, direct-to-PCR testing procedures, and colorimetric PCR-based viral detection methodologies have all been proposed to address the issue of access to accurate testing in a resource challenged setting [8–10].

Herein, the authors are utilizing a mechanical homogenization based direct-to-PCR viral detection method for SARS-CoV-2 detection from nasopharyngeal and oropharyngeal swabs to screen for both symptomatic and asymptomatic COVID-19 in Blantyre, Malawi. This homogenization based direct-to-PCR viral detection workflow was previously examined utilizing *in vitro* simulated nasopharyngeal swab samples spiked with clinically significant levels of human coronavirus 229E (HCoV-229E) for proof of concept testing [10]. In the initial study, the laboratory simulated samples processed using this novel methodology revealed a limit of detection of 1.2x10^3^ viral copies/mL with 96.30% sensitivity when detecting HCoV-229E [10].

While this technology has been reported as a proof of concept, *in vitro* observation, this brief manuscript examines the clinical feasibility and efficiency of this methodology [10]. In a head-to-head comparison of the direct-to-PCR testing and the traditional extraction-based PCR testing methods, asymptomatic and mild to moderately symptomatic patients were screened with both methods for SARS-CoV-2 detection.

## Materials and methods

### Sample collection

The upper respiratory tract samples used for this method validation study were obtained through the University of Malawi College of Medicine (Blantyre, Malawi). All samples were obtained with informed patient consent following the COVID-HCWEXPO protocols in IRB contract P.05/20/3053 approved by the Institutional Review Board of the University of Malawi College of Medicine (Blantyre, Malawi). Informed consent was obtained from all subjects involved in the study prior to participation. The study recruited participants over the age of 18 and consent was obtained via written consent by each participant and witnessed by the researcher collecting the sample.

Upper respiratory tract samples were obtained from a prospective cohort study to compare the risk of SARS-CoV-2 infection and exposure in healthcare workers compared to community members in Blantyre, Malawi between June and August 2020 (manuscript in preparation). 15 SARS-CoV-2 PCR positive and 15 SARS-CoV-2 PCR negative were randomly selected from the cohort of 300 adult participants. (Table 1). Patients presenting to the hospital with suspected severe COVID-19 in respiratory distress were excluded from this study.

**Table 1:** RT-qPCR results following the extraction-based and homogenization-based protocols represented as Ct values for each patient sample.

Sample	Extraction Ct Value	Homogenization Ct Value
1	22.02	26.73
2	23.8	34.78
3	25.31	30.57
4	25.36	34.57
5	25.71	25.4
6	26.58	27.56
7	27.12	28.13
8	27.28	28.49
9	27.35	27.75
10	27.67	31.8
11	31.36	32.95
12	34.14	36.64
13	38.0	45.0
14	38.49	45.0
15	38.58	45.0
16	45.0	37.09
17	45.0	45.0
18	45.0	45.0
19	45.0	37.41
20	45.0	45.0
21	45.0	45.0
22	45.0	45.0
23	45.0	45.0
24	45.0	45.0
25	45.0	45.0
26	45.0	45.0
27	45.0	45.0
28	45.0	45.0
29	45.0	37.36
30	45.0	45.0

At the time of sample collection, each patient provided two swab specimens, one nasopharyngeal swab and one oropharyngeal swab which were immediately stored in a single tube containing viral transport media to await further processing [5, 11]. Following sample collection, samples were stored at 4 C for up to 24 hr, if the sample was not going to be processed within the next 24 hr it was stored at -80 C until processing could occur.

### Extraction-based sample preparation

One mL of viral transport media was taken from the tube containing the nasopharyngeal and oropharyngeal swabs at room temperature and utilized in the Omega Bio-Tek Mag-Bind Viral DNA/RNA Kit in accordance with manufactures guidelines (Omega Bio-Tek, Cat. No. M6246). Final product of purified viral RNA was held on ice in preparation for PCR testing.

### Homogenization-based sample preparation

One mL of viral transport media was transferred from the original sample collection tube to a 2 mL screw cap tube (Omni International Inc, Cat. No. 19–647) and sealed. Sample tubes were then loaded into the Omni Bead Ruptor Elite (Cat. No. 19–647) for processing [10]. The samples were run at 4.2 m/s for 30 s, removed from the device, and allowed to sit for 60s following processing to allow for any forth in the tubes to settle [10].

### RT-qPCR detection of SARS-CoV-2

**Fig 1:**
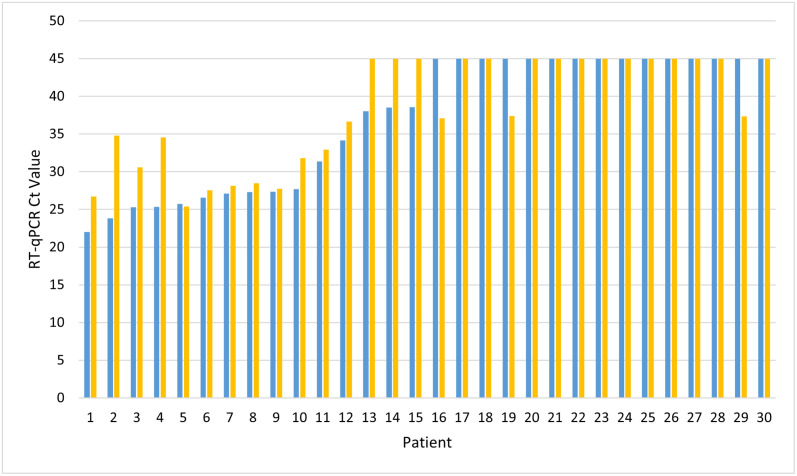
RT-qPCR results for SARS-CoV-2 detection utilizing the extraction-based or homogenization-based methods as demonstrated by paired Ct values. The blue bars depict Ct values following the extraction-based method and gold bars represent Ct values following the homogenization-based method.

Following sample processing with either the extraction-based or homogenization-based methods, CDC 2019-Novel Coronavirus (2019-nCoV) Real-Time RT-PCR Diagnostic Panel (Cat. No. 2019-nCoVEUA-01 1000 reactions) was used for detection of SARS-CoV-2. Briefly, 5μL of purified RNA or lysate, respectively was transferred into 15 μL reactions of Quantabio qScript XLT One-Step RT-qPCR ToughMix comprising of 3.5μL Nuclease-free Water, 1.5μL Combined Primer/Probe Mix, and 10μL qScript XLT One-Step RT-qPCR ToughMix (2X). The RT-qPCR reaction was premixed following vendor instructions and then loaded into the QuantiStudio 7 Flex Real Time PCR System (ThermoFisher Scientific, Cat. No. 4485698) and run with the vendor recommended temperature and cycle timing for a total of 45 amplification cycles. Cycle threshold (Ct) values were recorded and any sample with a Ct value less than or equal to 40 was labeled as positive for COVID-19 detection based on the US CDC recommended analysis of COVID-19 probe-based RT-qPCR results (Fig 1 and Table 1) [11].

### Statistical comparison of extraction and homogenization methodologies

**Fig 2:**
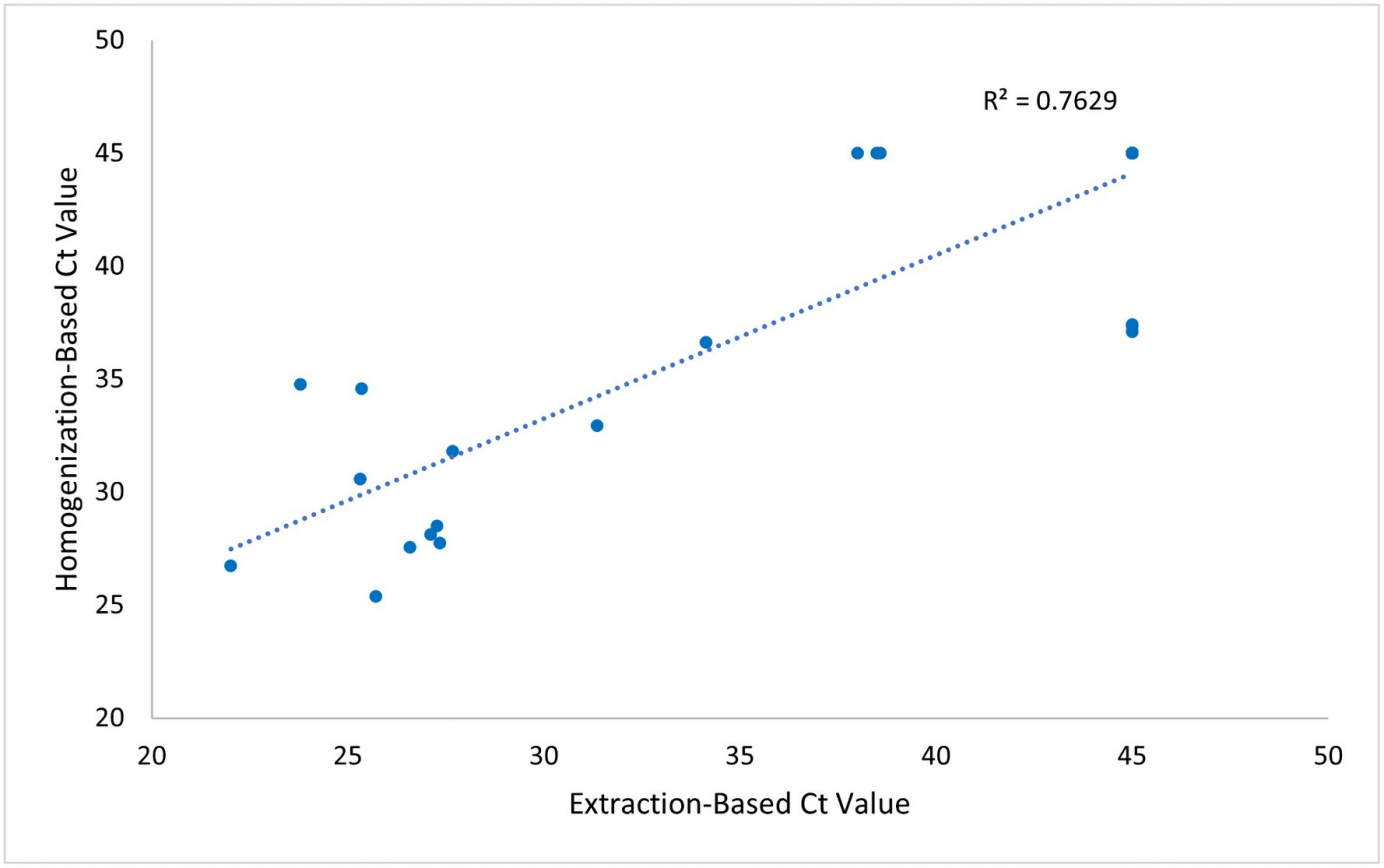
RT-qPCR results of SARS-CoV-2 plotting extraction-based Ct values versus homogenization-based Ct values for correlation evaluation. The best fit line and R^2^ value displayed demonstrate the positive relationship between both testing methodologies.

When comparing the extraction and homogenization-based sample preparation techniques described above, the final Ct values resulting from each method were paired under the original patient identification number for direct comparison. For the purpose of statistical analysis of the final results, negative samples where no amplification was detected in the presence of confirmed internal controls, were assigned a Ct value of 45.0. The positive predictive value (PPV) and negative predictive value (NPV) for the homogenization-based workflow were calculated using the extraction-based workflow as the gold standard method to compare against (Table 2). Additionally, the two resultant Ct values linked to the same patient from each testing method were plotted against each other for correlative visualization and a standard best fit line was produced to evaluate the R^2^ value (Fig 2).

**Table 2:** RT-qPCR results displayed as Ct values for patients with positive SARS-CoV-2 detection utilizing either the extraction-based or homogenization-based workflows.

Sample	Extraction Ct Value	Homogenization Ct Value	Positive Predictive Value		Negative Predictive Value	
1	25.71	25.4	100	80	100	50
2	22.02	26.73				
3	26.58	27.56				
4	27.35	27.75				
5	27.12	28.13				
6	27.28	28.49				
7	25.31	30.57				
8	27.67	31.8				
9	31.36	32.95				
10	25.36	34.57				
11	23.8	34.78				
12	34.14	36.64				
13	38.0	45.0				
14	38.49	45.0				
15	38.58	45.0				
16	45.0	37.09				
19	45.0	37.36				
29	45.0	37.41				

Positive predictive values and negative predictive values shown are calculated with the extraction-based workflow serving as the gold standard for reference. The PPV and NPV were also calculated following the proposed Ct cut off 37.0 for positives as well as the current 40.0 standard cut off for positive designation.

## Results

When directly comparing the capabilities of homogenization-based, direct-to-PCR workflow to traditional, extraction-based methodology for SARS-CoV-2 detection off nasopharyngeal and oropharyngeal swabs there was an overall 80% agreeability between both methods. A strong positive correlation was observed when plotting Ct values produced utilizing both examined methods linked to the same patient sample as demonstrated with an R^2^ of 0.763 (Fig 2). Except for one patient, all paired positive samples show a Ct value following homogenization-based testing to be greater than the extraction-based testing for the same patient (Fig 1 and Table 2). However, there were noted to be 6 positive samples that demonstrated disagreement following processing under both methodologies, where a sample was Ct positive for SARS-CoV-2 utilizing one method and not the other (Table 2). There was an equal distribution of disagreement between both testing methodologies with 3 of 30 patients testing positive for COVID-19 with extraction-based testing (patients 13, 14, and 15) and negative with the homogenization-based testing, and then the reverse with 3 of 30 patients testing positive for COVID-19 with homogenization-based testing (patients 16, 19, and 29) and negative with extraction-based testing (Table 2). These disagreements in SARS-CoV-2 detection were observed in samples which had results of Ct values greater than 37.08, indicating a low viral load (Tables 1 and 2). When comparing Ct values below 37.08, there was a 100% agreement for detection of SARS-CoV-2 utilizing both methodologies (Table 2).

## Discussion

In this clinical validation study, we utilized 30 paired nasopharyngeal and oropharyngeal swab samples participants in a prospective cohort study to assess the occupational risk of SARS-CoV-2 infection or exposure in Blantyre, Malawi to examine the efficacy of a direct-to-PCR, homogenization-based workflow when compared to the gold standard extraction-based workflow for detection of SARS-CoV-2. 80% agreeability between all samples following processing was noted, with a positive direct correlation between the resultant Ct values produced when processing each sample with both methods. An R^2^ of 0.763 and positive predictive value of 80% demonstrate a moderate level of acceptability for implementing the proposed direct-to-PCR method into clinical practice. Additionally, when moving the negative detection cut off Ct value from 40.0 to 37.0 the improvement of both the positive predictive value and negative predictive value to 100% and agreement of 100% between both methodologies warrants further investigation while strengthening the argument for clinical implementation.

Throughout the progression of the COVID-19 global pandemic, the need for efficient and effective diagnostic testing has proven paramount to the global response, with PCR based viral detection off swabs serving as the current gold standard for testing [2–4]. However, the traditional extraction-based methodology detailed in this manuscript requires many chemical reagents to complete and has become financially burdensome secondary to strain placed on the global supply chains for many of these reagents and consumable plastics. The increase in demand for the supplies needed to complete the traditional extraction-based method coupled with the limited global supply for the reagents created a scenario where many wealthy countries and communities were able to purchase all of the supplies needed for testing, leaving resource challenged settings struggling to ensure availability of timely and accurate viral detection methods.

In addition to the high cost associated with the accurate extraction-based workflow, the total run time for each sample to be processed in this method can take up to 3 hours from extraction to PCR results when using a fully automated workflow. When receiving thousands of samples per day, a backlog can easily be created utilizing the extraction-based method causing delays in relaying critical positive or negative results to patients and local health officials. Developing approaches to reduce the total time per test while maintaining accuracy also emerged as an area of needed innovation in response to the COVID-19 pandemic.

To address these limitations in viral testing many approaches have been proposed ranging from point of care, lateral flow antigen tests to rapid PCR tests that offer solutions on either time or cost associated with testing [6–8]. While many of the proposed technologies for increasing testing either reduce time or reduce required reagents, they often do not achieve both while maintaining diagnostic accuracy similar to the current extraction-based PCR workflow [6, 7]. Herein, the validation of the proposed direct-to-PCR, homogenization-based methodology offers a solution through reducing the time and reagents needed to process each sample while maintaining an acceptable level of accuracy when compared to the extraction-based method.

Of note, the demonstrated agreeability between the direct-to-PCR and traditional methods displayed a pattern of all except for one of the SARS-CoV-2 positive samples had a lower Ct value following the extraction protocol versus the homogenization protocol. This is representative of the PCR inhibitors present in the lysate following homogenization that are not removed due to the lack of chemical purification steps when compared to the extraction protocols. Given the presence of inhibitors, it is expected that the Ct values are greater than the purified samples from kit-based extractions. However, the high agreeability in detecting SARS-CoV-2 between the methods demonstrates that these inhibitors remaining in the lysate transferred to the RT-qPCR in the homogenization method do not prohibit adequate RNA amplification for detection. This pattern confirmed with a strong R^2^ value when plotting Ct values from both methods against each other supports the reliability of the direct-to-PCR workflow when dealing with the variability of viral loads in a clinical sample.

While we acknowledge that this initial clinical validation study demonstrated inconsistent detection when the Ct values were greater than 37, the 100% positive predictive value and 100% negative predictive value at Ct values less than 37 qualifies this methodology for further large-scale evaluations (Table 2). It has been documented that between commercially available kits there is decreasing agreement on SARS-CoV-2 detection as Ct values grow past 36 [12–14]. The authors acknowledge that this manuscript has its limitations in statistical robustness and the limited data it provides, such as the lack of technical replicates or viral load quantifications. However, we felt it was prudent to disseminate the limited information we have developed around this novel process for viral detection in an effort to assist in the current international need for COVID-19 testing innovation and improvement. It is our hope that this efficient and cost-effective measure for COVID-19 PCR based testing can work to fill the void in testing in many areas of the world.

Using the small mechanical footprint of a bench top homogenizer and a standard thermocycler, and without the need of a multitude of chemical reagents, the direct-to-PCR workflow can be set up almost anywhere. The ease of system configuration and simple operational steps needed to complete this workflow support the adaptability of this novel method to be implemented in any area, including resource challenged settings without the funds or laboratory spaces to support fully automated extraction based viral testing systems. It is critical that we as a global community continue to commit to the development of testing strategies to assist in COVID-19 public health efforts in all areas of the world if we are going to implement successful pandemic mitigation efforts.

Given the high levels of agreeability between the observed workflows, in conjunction with the significant reduction in cost and processing time by utilizing a 30 second mechanical homogenization step versus an hour-long reagent heavy extraction procedure, this direct-to-PCR methodology has potential to be further incorporation into global COVID-19 detection strategies. Herein, we believe our novel direct-to-PCR workflow is a potential solution to increase access to efficient SARS-CoV-2 detection in resource challenged areas.
